# In Vitro Comparative Study of Near-Infrared Photoimmunotherapy and Photodynamic Therapy

**DOI:** 10.3390/cancers15133400

**Published:** 2023-06-28

**Authors:** Susumu Yamashita, Miho Kojima, Nobuhiko Onda, Makoto Shibutani

**Affiliations:** 1 Laboratory of Veterinary Pathology, Division of Animal Life Science, Institute of Agriculture, Tokyo University of Agriculture and Technology, 3-5-8 Saiwai-cho, Fuchu 183-8509, Tokyo, Japan; susumu.yamashita@olympus.com; 2 Medical Evaluation Engineering, Olympus Medical Systems Corporation, 2-3 Kuboyama-cho, Hachioji 192-8512, Tokyo, Japan

**Keywords:** near-infrared photoimmunotherapy (NIR-PIT), monoclonal antibody (mAb), IRDye700, photodynamic therapy (PDT), talaporfin sodium

## Abstract

**Simple Summary:**

Near-infrared photoimmunotherapy (NIR-PIT) is a newly developed cancer treatment that uses photoreactive agents and light irradiation, while photodynamic therapy (PDT) is an existing phototherapy using photosensitizers and light irradiation as well as NIR-PIT. The current study investigated the properties of these therapies and their differences with a focus on their cellular binding/uptake specificity and cytotoxicity in vitro. NIR-PIT showed molecule-selective responses and cytotoxicity, whereas PDT showed non-selective cell type responses and cytotoxicity. Additionally, NIR-PIT and PDT induced damage-associated molecular patterns (DAMPs), a surrogate marker of immunogenic cell death, although PDT had different sensitivity between cell lines. Therefore, molecule-specific NIR-PIT may have advantages compared with PDT in terms of efficiency in tumor visualization and induction of DAMPs.

**Abstract:**

Near-infrared photoimmunotherapy (NIR-PIT) is a new phototherapy that utilizes a monoclonal antibody (mAb) against cancer antigens and a phthalocyanine dye, IRDye700DX (IR700) conjugate (mAb-IR700). Photodynamic therapy (PDT) is a combination therapy that utilizes photoreactive agents and light irradiation as well as NIR-PIT. In the present study, we compared these therapies in vitro. The characterization of cellular binding/uptake specificity and cytotoxicity were examined using two mAb-IR700 forms and a conventional PDT agent, talaporfin sodium, in three cell lines. As designed, mAb-IR700 had high molecular selectivity and visualized target molecule-positive cells at the lowest concentration examined. NIR-PIT induced necrosis and damage-associated molecular patterns (DAMPs), a surrogate maker of immunogenic cell death. In contrast, talaporfin sodium was taken up by cells regardless of cell type, and its uptake was enhanced in a concentration-dependent manner. PDT induced cell death, with the pattern of cell death shifting from apoptosis to necrosis depending on the concentration of the photosensitizer. Induction of DAMPs was observed at the highest concentration, but their sensitivity differed among cell lines. Overall, our data suggest that molecule-specific NIR-PIT may have potential advantages compared with PDT in terms of the efficiency of tumor visualization and induction of DAMPs.

## 1. Introduction

Near-infrared photoimmunotherapy (NIR-PIT) is a new phototherapy for cancer that involves the use of a conjugate, a phthalocyanine dye called IRDye700DX (IR700), which is covalently bound to a monoclonal antibody (mAb) against cancer antigens [1,2]. The conjugate selectively binds to cancer antigens that are overexpressed on the cell membrane and induces rapid cell death when exposed to light of approximately 690 nm [3,4,5]. This therapy is a molecular targeted therapy, and NIR-PIT targeting various cancer antigens was reported to have highly therapeutic effects on various cancers [6,7,8,9]. Therefore, NIR-PIT is expected to be effective against any cancer that overexpresses a cancer antigen, regardless of the cancer type, if antibodies against the cancer antigen are available. Currently, a global clinical trial in phase 3 is underway to investigate the efficacy of ASP-1929, an mAb-IR700 that targets the epidermal growth factor receptor (EGFR), in patients with recurrent or inoperable head and neck cancer (NCT03769506). In Japan, ASP-1929 received conditional approval for clinical use as a first-in-class drug in September 2020 [10,11].

Photodynamic therapy (PDT) is a cancer treatment that combines a photosensitizer and light irradiation at a specific wavelength. Several photosensitizers are currently used in clinical applications [12,13]. Among them, talaporfin sodium (mono-L-aspartyl chlorine e6, NPe6) is a second-generation photosensitizer used for PDT. Talaporfin sodium is taken up into tumor cells by endocytosis and accumulates in lysosomes [14,15]. Laser irradiation at 664 nm, the Q band absorption peak wavelength of talaporfin sodium, induces cell death through the collapse of lysosomes and the release of hydrolytic enzymes into the cytosol [16,17]. In Japan, talaporfin sodium was approved for early-stage lung cancer in 2003 [18], primary malignant brain tumors in 2013 [19], and localized residual recurrent esophageal cancer after chemoradiation or radiation therapy in 2015 [20].

The cancer therapies NIR-PIT and PDT have the use of photoreactive agents and light-irradiation devices in common. However, there are some differences. For example, in contrast to PDT, NIR-PIT has cancer cell selectivity related to the use of mAb against cancer antigens. The mechanisms involved in cell death by NIR-PIT were recently reported [21,22,23], and it is proposed that a major mechanism of NIR-PIT is physical destruction of the cell membrane, which is caused by photoinduced physicochemical changes to mAb-IR700 associated with the release of ligands from IR700. This contrasts with the physiological cell death induced by PDT, which relies on damage to intracellular organelles by singlet oxygen and other secondary molecules such as reactive oxygen species produced by the excitation of photosensitizers [24]. The principal consequence of NIR-PIT is necrosis [7], whereas PDT can cause a variety of cell death mechanisms including apoptosis, paraptosis, necrosis, and autophagy [25]. However, NIR-PIT and PDT were reported to induce the activation of anti-tumor host immunity by causing immunogenic cell death (ICD) [26,27,28,29]. We believe that it is important to compare NIR-PIT and the existing technology PDT in the same study for the accurate interpretation of the therapeutic usefulness of NIR-PIT and its optimization for widespread clinical use; however, to the best of our knowledge, no comparative studies on the cell death mechanism of NIR-PIT and PDT have been reported.

The present study compared NIR-PIT and PDT using talaporfin sodium (TS-PDT) in vitro. Two mAb-IR700 forms for NIR-PIT and talaporfin sodium for PDT were examined in terms of their cellular binding/uptake and target selectivity using three cell lines. Then, concentration-related cytotoxic effects induced by light irradiation were examined. Furthermore, patterns of cell death as well as ICD markers of NIR-PIT and TS-PDT were investigated.

## 2. Materials and Methods

### 2.1. Reagents

A water-soluble, silica-phthalocyanine derivative, IRDye700DX NHS ester, was obtained from LICOR Biosciences (Lincoln, NE, USA). Panitumumab (Vectibix), a fully humanized IgG2 mAb directed against human EGFR, was purchased from Takeda Pharmaceutical Co. Ltd. (Osaka, Japan). Trastuzumab (Herceptin), a 95% humanized IgG1 mAb against the extracellular domain of HER2, was purchased from Chugai Pharmaceutical (Tokyo, Japan). Talaporfin sodium was obtained from MedChemExpress (HY-16477; Princeton, NJ, USA).

### 2.2. Synthesis of IR700-Conjugated mAbs

The conjugation of IR700 with antibodies was performed according to a previous report [7], with some modifications. Briefly, panitumumab or trastuzumab (6.8 nmol) was incubated with IR700 (25 nmol) in 0.1 M Na_2_HPO_4_ (pH 8.5) at room temperature for 1 h. The reaction mixture was purified with an ultrafiltration column (Amicon Ultra 30K; Millipore, Merck, Darmstadt, Germany) to derive panitumumab-IR700 (Pan-IR700) and trastuzumab-IR700 (Tra-IR700). The protein concentration and an average of three IR700 molecules bound to a single antibody were confirmed by measuring absorption at 280 nm and 689 nm using a spectrometer (NanoDrop One; Thermo Fisher Scientific, Waltham, MA, USA). Following the manufacturer’s protocols, calculations were made to determine the antibody-IR700 conjugate concentration and the number of IR700 molecules per antibody molecule. The concentration mentioned for the mAb-IR700 in this study was the antibody-IR700 conjugate concentration.

### 2.3. Cell Culture

The human epidermis carcinoma cell line A431 (ECACC85090402) was obtained from the European Collection of Authenticated Cell Cultures through DS Pharma Biomedical (Osaka, Japan). The mouse embryo fibroblast cell line BALB/3T3 clone A31 (JCRB9005) was obtained from the Japanese Collection of Research Bioresources Cell Bank (Osaka, Japan). The human breast ductal carcinoma cell line BT-474 (HTB-20) was obtained from the American Type Culture Collection through Summit Pharmaceuticals International Corporation (Tokyo, Japan). These cell lines were cultured in Dulbecco’s modified Eagle’s medium (10569010; Thermo Fisher Scientific) supplemented with 10% fetal bovine serum (26140079; Thermo Fisher Scientific) and 1% penicillin/streptomycin (15140122; Thermo Fisher Scientific). Each cell line was cultured in tissue culture flasks at 37 °C in a 5% CO_2_ humidified atmosphere.

### 2.4. Live Cell Imaging

To examine the cellular binding/uptake specificity and distribution of mAb-IR700 or talaporfin sodium, live cell fluorescence imaging was performed using cultured cells incubated with mAb-IR700 or talaporfin sodium. Cells were seeded onto 24-well glass bottom plates and incubated for 24 h. The culture medium was replaced with fresh medium containing talaporfin sodium or mAb-IR700, and incubated for 1, 4, and 24 h at 37 °C. Then, the cells were washed with PBS, and the nuclei were stained with Hoechst 33342 (Thermo Fisher Scientific). Cell images were captured using an inverted fluorescence microscope (IX-83; Olympus, Tokyo, Japan) equipped with a fluorescence light source (U-HGLGPS, Olympus) and a monochrome/color camera (DP80; Olympus). For the fluorescence imaging of IR700 and talaporfin sodium, we used a Cy5 filter cube (Cy5-4040C: 628/40 nm excitation, 692/40 nm emission, and 660 nm dichroic mirror; Semrock Inc., Rochester, NY, USA), and captured images under identical conditions. Obtained fluorescence images of IR700 and talaporfin sodium were analyzed by Image J software (National Institutes of Health, Bethesda, MD, USA). The mean fluorescence intensity (MFI) of IR700 and talaporfin sodium was calculated from three microscopic fields. All experiments were repeated three times.

### 2.5. In Vitro NIR-PIT and TS-PDT

The parameters involved in NIR-PIT and TS-PDT (e.g., agent concentration and incubation time) were determined with reference to previous reports [30,31,32,33]. Cells were seeded onto 96-well plates and incubated for 24–48 h. The cells were incubated with mAb-IR700 for 1 h or talaporfin sodium for 4 h in culture medium. After washing with PBS, cells incubated with mAb-IR700 were irradiated with 690 nm light from a continuous wave laser (BWF1-690-300-E; B&W TEK, Newark, DE, USA), and cells incubated with talaporfin sodium were irradiated with 670 nm light from a continuous wave laser (BWF1-670-300-E; B&W TEK). The power density was adjusted to 50 mW/cm^2^ using a radiometer (PD300-BB-50mW; OPHIR Photonics, Jerusalem, Israel), and the irradiation was performed for 200 s (the energy density was 10 J/cm^2^).

### 2.6. In Vitro Cytotoxicity Assay

To examine the immediate cytotoxicity of NIR-PIT and TS-PDT, we performed three assays. The time course imaging of cell morphological changes was performed using an IX-83 inverted microscope for 15 min, from before the start of irradiation to 10 min after the end of irradiation. To detect the rupture of cell membranes within 60 min after irradiation, calcein-AM/propidium iodide (PI) double-staining was performed according to a previous report [26]. Cells were incubated with fresh medium containing calcein-AM (200 nM; PromoKine, Heidelberg, Germany) and PI (500 nM; Thermo Fisher Scientific) for 15 min at room temperature. Then, time course imaging was performed using an IX-83 inverted microscope, from before the start of irradiation to 1 h after the end of irradiation, without medium changes. The cytotoxic effects of NIR-PIT and TS-PDT on cultured cells were assessed 1 day after irradiation using Hoechst/PI double-staining and a cytotoxicity lactate dehydrogenase (LDH) assay. For Hoechst/PI double-staining, cells were incubated with fresh medium containing PI (500 nM; Thermo Fisher Scientific) and Hoechst (5 µg/mL) for 10 min at room temperature and washed twice with PBS. Fluorescence images were captured using an IX-83 microscope. An LDH assay was performed with culture supernatants using a Cytotoxicity LDH Assay Kit-WST (Dojin Chemicals, Kumamoto, Japan). In accordance with the manufacturer’s protocols, the measurement of absorbance at 490 nm was performed by a microplate reader (Nivo S; PerkinElmer, Waltham, MA, USA) and the cytotoxicity rate was calculated.

### 2.7. Annexin V/PI Staining

To examine the patterns of cell death induced by NIR-PIT and TS-PDT, annexin V/PI staining was performed. When staining cells with annexin V-FITC and PI, necrotic cells are stained with PI and annexin V-FITC, whereas apoptotic cells are stained only with annexin V-FITC. Cells were seeded onto 24-well glass bottom plates and incubated for 48 h. The cells were treated with NIR-PIT or TS-PDT and stained with a MEBCYTO Apoptosis Kit (MBL Co., Nagoya, Japan) at 3 h after treatment in accordance with the manufacturer’s protocols. Fluorescence images were captured using an IX-83 microscope.

### 2.8. Detection of ICD Markers

To investigate the potential of ICD in NIR-PIT and TS-PDT, we analyzed extracellular ATP and high-mobility group protein B1 (HMGB1) localization among damage-associated molecular patterns (DAMPs), which are known ICD markers. For the quantitative analysis of extracellular ATP, cells were seeded onto 24-well glass bottom plates and incubated for 48 h. Before TS-PDT or NIR-PIT, the cells were washed with PBS and the medium was replaced with phenol red-free culture medium. Culture supernatant was collected 1 h after NIR-PIT or TS-PDT and then extracellular ATP was quantified by a luciferin-based ATP assay (ENLITEN; Promega, Madison, WI, USA). Luminescence signals were measured by a microplate reader (Nivo S; PerkinElmer). Cells exposed to irradiation only were used as controls. Fluorescent immunostaining was performed for the analysis of HMGB1 localization. Cells were seeded onto 8-well chamber slides and incubated for 48 h, followed by treatment with NIR-PIT or TS-PDT, and then fixation with 4% paraformaldehyde and permeabilization with 0.1% Triton X-100 at 1 h after treatment. Cells were then blocked with 5% goat serum (S-1000; Vector Labs., Burlingame, CA, USA) in PBS for 1 h at room temperature. Next, the cells were incubated with anti-HGMB1 antibody (ab18256; Abcam, Cambridge, MA, USA) overnight at 4 °C, washed with PBS, and stained with Alexa Fluor-conjugated anti-rabbit IgG (A21206; Invitrogen) for 30 min at room temperature. Stained cells were mounted with hard-set medium containing NucBlue stain (P36981; Invitrogen), and cell images were captured using a laser scanning confocal microscope (FV3000; Olympus). HMGB1 translocation was assessed by measuring the fluorescence intensity of nuclear HMGB1. Details of the procedure are described in the Materials and Methods section of the Supplementary Materials.

### 2.9. Statistical Analysis

Numerical data are presented as the mean ± SD. Numerical data were assessed using Tukey’s test after verification of the homogeneity of the variances by Bartlett’s test. The Steel–Dwass test was used for heterogeneous data. *p*-values less than 0.05 were considered statistically significant.

## 3. Results

### 3.1. Cellular Binding/Uptake Specificity and Distribution of mAb-IR700 and Talaporfin Sodium

**Figure 1:**
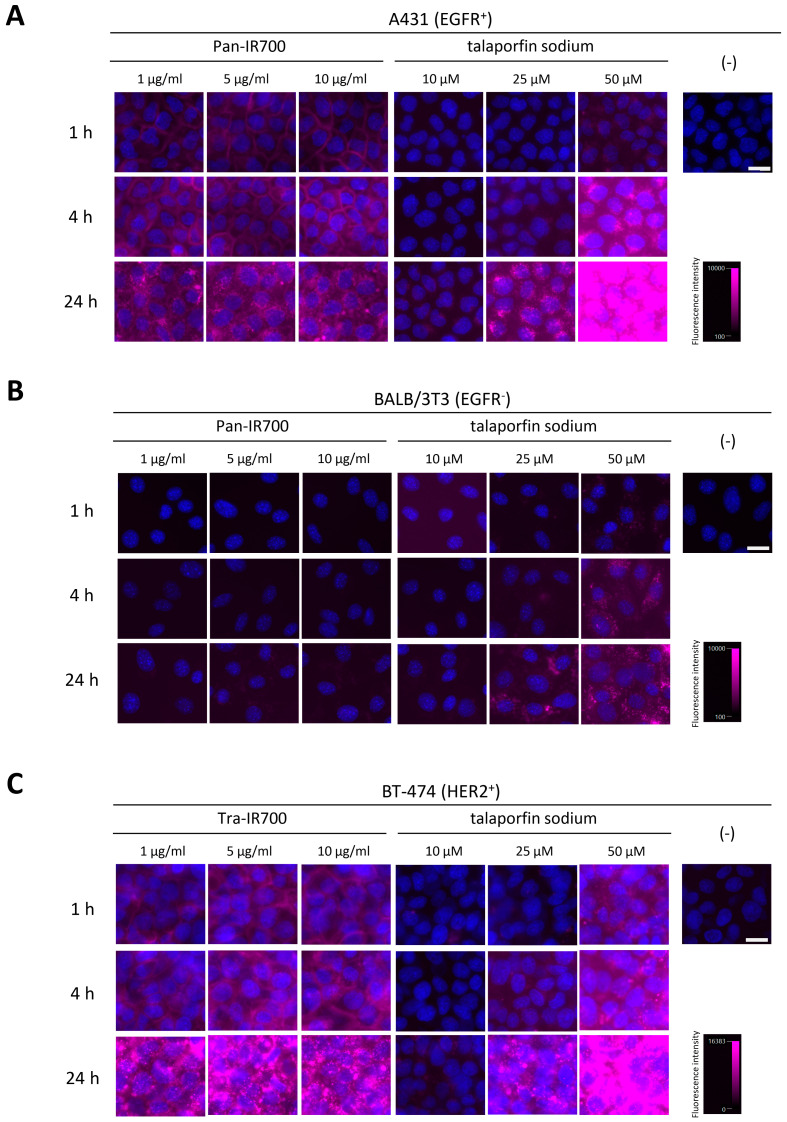
Cellular binding/uptake specificity and distribution of mAb-IR700 and talaporfin sodium. Fluorescence images of live cells stained for nuclei (blue) after incubation with mAb-IR700 or talaporfin sodium (magenta). (**A**) A431 cells, (**B**) BALB/3T3 cells, and (**C**) BT-474 cells. Scale bar = 20 µm.

The cellular binding/uptake specificity and distribution of mAb-IR700 and talaporfin sodium in three cell lines were analyzed by fluorescence microscopy. No autofluorescence signals were observed when the cell lines were not incubated with mAb-IR700 or talaporfin sodium (Figure 1). Pan-IR700 and Tra-IR700 showed clear fluorescence signals only in the EGFR-positive cell line, A431, and HER2-positive cell line, BT-474, respectively (Figure 1 and Figure S1B). The increase in MFI of mAb-IR700 was dependent upon the incubation time, but not on the mAb-IR700 concentration (Figure S1A). Furthermore, the fluorescence signals localized at the cell membrane after 1 h incubation, whereas the positive signals were mainly observed in the cytoplasm after 24 h incubation. In contrast, the MFI of talaporfin sodium was increased in all three cell lines and was dependent on the incubation time and talaporfin sodium concentration (Figure 1 and Figure S1A).

### 3.2. Cytotoxicity of NIR-PIT and TS-PDT

**Figure 2:**
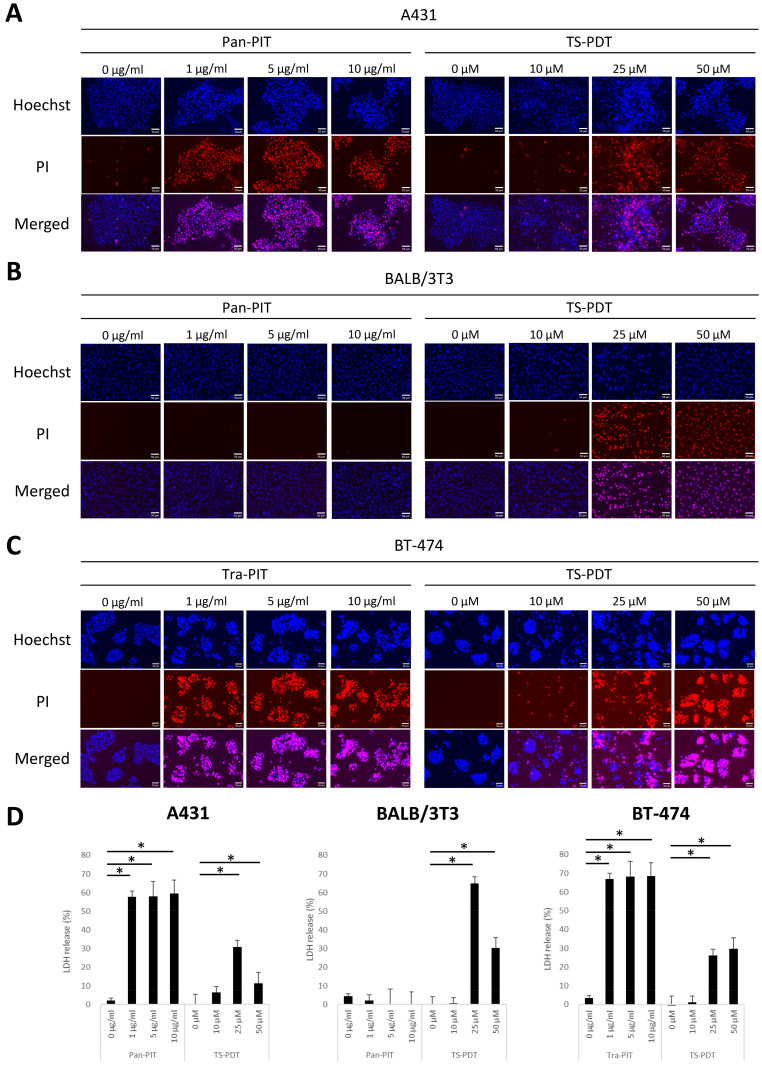
Evaluation of cell death induced by NIR-PIT and TS-PDT. Hoechst and PI double-stained images 1 day after irradiation. (**A**) A431 cells, (**B**) BALB/3T3 cells, and (**C**) BT-474 cells. Scale bar = 50 µm. (**D**) LDH assay at 1 day after irradiation in the three cell lines. Data are presented as means ± SD (n = 3; Tukey’s test; * *p* < 0.05).

Based on the characterization of the cellular binding/uptake and distribution, the drug-light interval between the starting time point of incubation with photoreactive agents and laser irradiation was determined to be 1 h for mAb-IR700 and 4 h for talaporfin sodium. Hoechst/PI double-staining showed that the number of PI-positive dead cells was increased in A431 cells by panitumumab-based NIR-PIT (Pan-PIT) and in BT-474 cells by trastuzumab-based NIR-PIT (Tra-PIT) (Figure 2A,C). This increase in the number of dead cells induced by NIR-PIT was observed at all concentrations examined. However, Pan-PIT did not increase the number of dead cells in BALB/3T3 cells (Figure 2B). In contrast, the number of PI-positive dead cells was increased in all cell lines 1 day after TS-PDT at > 25 µM compared with the number of cells receiving light irradiation alone (Figure 2A–C). There was no toxicity induced by light irradiation alone in all cell lines examined. NIR-PIT and TS-PDT cytotoxicity were indicated by LDH release in accordance with the PI-positive fluorescence signals (Figure 2D).

### 3.3. Patterns of Cell Death Induced by NIR-PIT and TS-PDT

**Figure 3:**
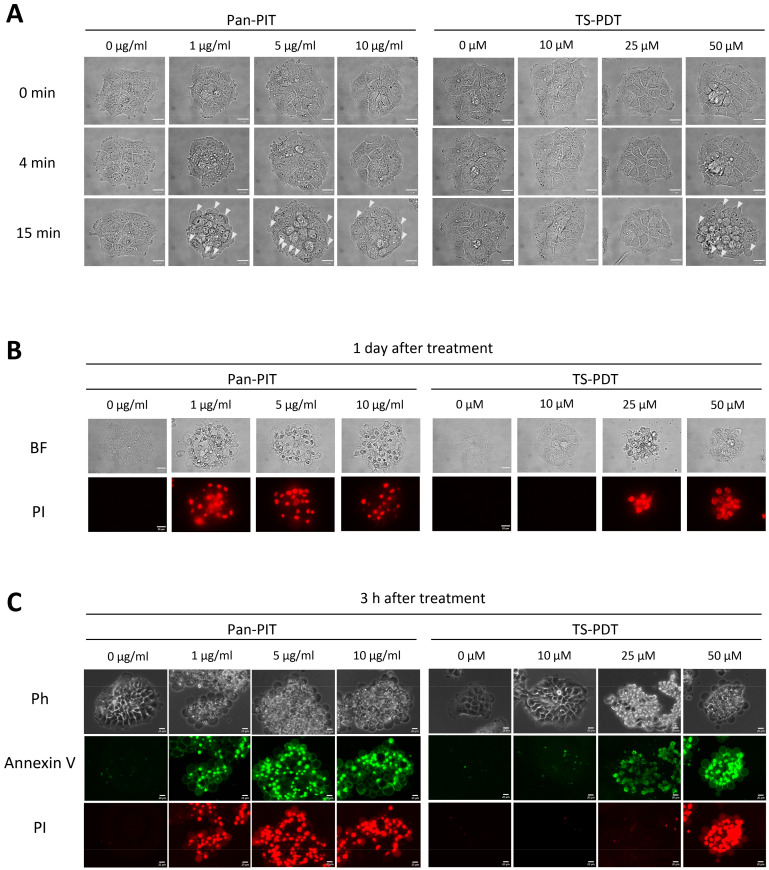
Evaluation of pattern of cell death induced in A431 cells by NIR-PIT and TS-PDT. (**A**) Time course images during irradiation. 0 min: before irradiation; 4 min: immediately after irradiation; 15 min: approximately 10 min after irradiation. White arrow indicates blebbing. Scale bar = 20 µm. (**B**) Bright-field images and PI fluorescence images at 1 day after irradiation. Scale bar = 20 µm. (**C**) Phase contrast images and annexin V-FITC/PI fluorescence images at 3 h after irradiation. Scale bar = 20 µm.

Morphological changes of cultured cells after NIR-PIT or TS-PDT were next observed. A431 cells treated with Pan-PIT showed cell swelling and blebbing on the cell membrane 15 min after irradiation; however, these morphological changes were not observed in A431 cells that received light irradiation alone, or in BALB/3T3 cells treated with Pan-PIT. Furthermore, A431 and BALB/3T3 cells incubated with 50 µM talaporfin sodium showed cell swelling and blebbing of the plasma membrane at 15 min after irradiation, but these morphological changes were not observed in A431 and BALB/3T3 cells treated with TS-PDT at < 50 µM (Figure 3A and Figure S2). Furthermore, A431 cells treated with the highest concentration of Pan-PIT or TS-PDT showed decreased calcein fluorescence at 30 min after irradiation (Figure S3). However, A431 cells incubated with 25 µM talaporfin sodium showed cell death with cell aggregation and fragmentation 1 day after irradiation, even though morphological changes were not observed immediately after irradiation (Figure 3B). In the examination of patterns of cell death using annexin V-FITC and PI staining 3 h after Pan-PIT or TS-PDT, A431 cells treated with Pan-PIT were consistently annexin V-positive and PI-positive, whereas A431 cells treated with TS-PDT showed multiple staining patterns including annexin V negativity and PI negativity at 10 µM, annexin V positivity and PI negativity at 25 µM, and annexin V positivity and PI-positivity at 50 µM, respectively (Figure 3C).

### 3.4. Analysis of ICD Markers following NIR-PIT and TS-PDT

**Figure 4:**
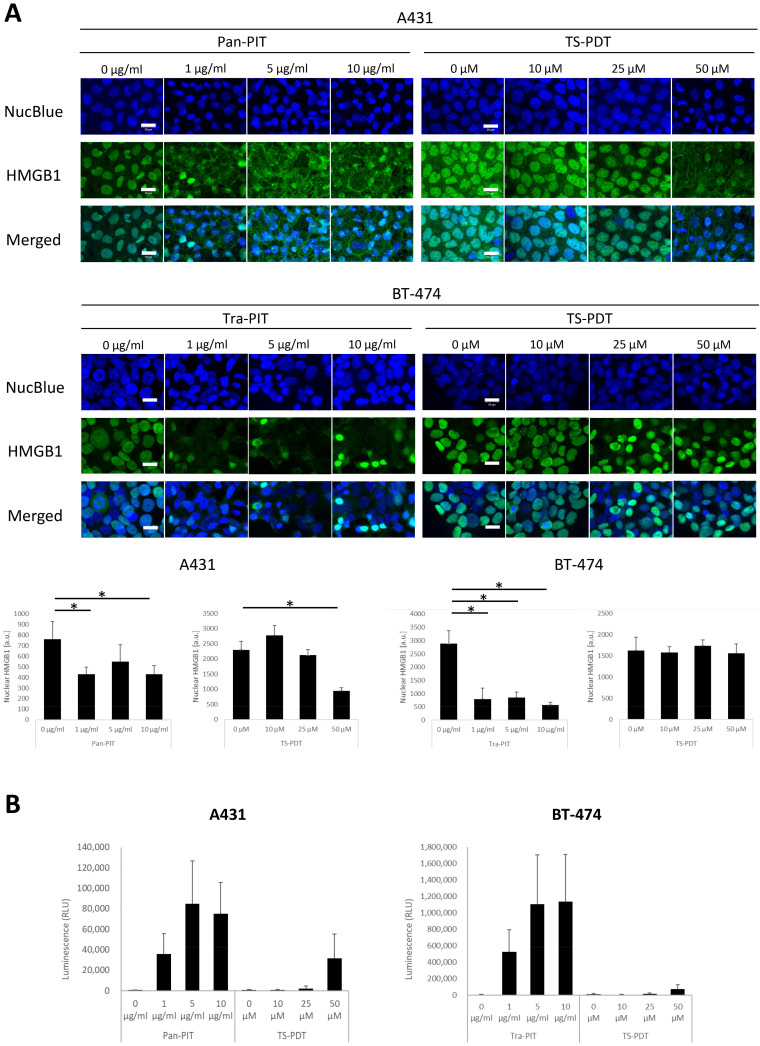
Analysis of ICD markers following NIR-PIT or TS-PDT. (**A**) Confocal microscopy images of HMGB1 and quantification of HMGB1 translocation. HMGB1 was stained at 1 h after treatment of A431 and BT-474 cells with NIR-PIT or TS-PDT. Scale bar = 20 µm. Fluorescence intensity of nuclear HMGB1 was quantified; data are presented as means ± SD from five microscopic fields (Tukey’s test; * *p* < 0.05). (**B**) ATP assays of culture supernatants. Data are presented as means ± SD (n = 3; Steel–Dwass test).

HMGB1 translocation and extracellular ATP release were analyzed 1 h after NIR-PIT or TS-PDT. HMGB1 staining showed the disappearance of nuclear HMGB1 in A431 and BT-474 cells treated with NIR-PIT at ≥ 1 µg/mL. The loss of nuclear HMGB1 in A431 cells was also observed with TS-PDT at 50 µM, but not at lower concentrations in this cell line or at all concentrations examined in BT-474 cells (Figure 4A). Similar results were obtained with the ATP assay. NIR-PIT at all concentrations induced extracellular ATP increase in A431 and BT-474 cells. TS-PDT at 50 µM also induced extracellular ATP increase in A431 cells, but not at lower concentrations in this cell line or at all concentrations examined in BT-474 cells (Figure 4B).

## 4. Discussion

In the current study, we examined the cellular binding, uptake, and specificity of mAb-IR700 (Figure S5A) and talaporfin sodium (Figure S5B) in three cell lines: A431, BALB/3T3, and BT-474. The binding of two mAb-IR700 forms (targeting EGFR or HER2) to target molecules at the cell membrane was observed, and a target-molecule-negative cell line did not show mAb-IR700 fluorescence signals. We found target-molecule-bound mAb-IR700 was internalized into cells in a time-dependent manner, indicating that it can bind specifically to cancer cells via cell surface antigens and traffic into the cytoplasm, consistent with other antibody–drug conjugates [34]. In contrast, talaporfin sodium was taken up by all cell lines examined in the current study. The fluorescence signals of talaporfin sodium showed a granular pattern in the cytoplasm. This uptake increased in a concentration- and time-dependent manner, consistent with previous reports [35,36]. These results suggest that talaporfin sodium is taken up by a non-selective mechanism for cell type. In addition, we showed that mAb-IR700 could visualize target-molecule-positive cells, even at a concentration of 1 µg/mL, which is equivalent to 6.8 nM. In contrast, fluorescence signals were detected in cells incubated with talaporfin sodium at ≥25 µM. These results indicate that mAb-IR700 can efficiently visualize target cells at doses greater than 1000-fold lower than talaporfin sodium under the current imaging conditions.

Light irradiation of cells expressing EGFR or HER2 at the cell membrane that were incubated with mAb-IR700 induced cell death at all concentrations. The mechanism of this cytotoxic effect was related to NIR-PIT, a highly molecule-selective therapy that requires both mAb-IR700 binding to the cell membrane and light irradiation to cause cytotoxicity, as reported previously [1,7]. However, TS-PDT induced cytotoxicity in cells that took up talaporfin sodium. TS-PDT requires both the uptake of talaporfin sodium and light irradiation to cause cytotoxicity, as reported previously [33,35]. Cytotoxicity was observed in all cell lines examined, suggesting that the cell specificity of cytotoxicity induced by TS-PDT may be dependent on the cellular uptake capability of talaporfin sodium. Taken together, both NIR-PIT and TS-PDT were cytotoxic under each optimal condition, and this cytotoxicity correlated with the amount of agent fluorescence positivity. These results suggest that the fluorescence signals of mAb-IR700 or talaporfin sodium in target cells may be a parameter that could be used to assess the treatment efficacy of NIR-PIT or TS-PDT.

Our previous study revealed that BT-474 cells treated with Tra-IR700 underwent necrosis with rapid morphological changes through light irradiation [7]. In the present study, A431 cells treated with Pan-PIT showed swelling and blebbing at 4 and 15 min after light irradiation, respectively. These morphological changes were observed regardless of the concentration of Pan-IR700. In addition, NIR-PIT showed a decrease in calcein fluorescence at 30 min after irradiation. These results suggest that NIR-PIT initially induced cell swelling and blebbing, followed by rupture of the cell membrane. The cells treated with NIR-PIT were dead 1 day after irradiation, and analysis at 3 h after NIR-PIT revealed that these cells were annexin V positive and PI positive. These results suggest that NIR-PIT induces necrosis regardless of the mAb-IR700 concentration. TS-PDT at 50 µM in A431 cells induced the same morphological changes and decrease in calcein fluorescence, as observed in cells immediately after NIR-PIT. A previous study reported that the extracellular photosensitizing reaction of talaporfin sodium causes bleb formation, which eventually ruptures the cell membrane leading to necrosis [37]. In the current study, the morphological changes induced by TS-PDT were observed at 50 µM only and might have been induced by a photosensitizing reaction in the vicinity of the cell membrane. Thus, TS-PDT at 50 µM may have had a similar cytotoxic effect to NIR-PIT by causing rapid cell membrane damage rather than damage to intracellular organelles, which is the main mechanism of TS-PDT. In contrast, cell death occurred at 25 µM 1 day later. At this concentration, dead cells showed morphological characteristics that differed from those of the burst cells observed with 50 µM TS-PDT, similarly to the NIR-PIT-induced cell death morphology, including shrinkage and vesicle formation. Annexin V/PI staining at 3 h after light irradiation revealed that 25 and 50 µM TS-PDT differentially induced apoptosis and necrosis, respectively, consistent with a previous study [38]. Our study results suggest that the pattern of cell death induced by TS-PDT is a concentration-dependent shift from apoptosis to necrosis. In summary, NIR-PIT consistently induced necrosis in targeted cells at all concentrations examined, whereas TS-PDT induced cell death at ≥ 25 µM, and the pattern of cell death varied with the concentration of talaporfin sodium.

ICD is induced by the release of spatiotemporally regulated immunogenic signals. Such signals are conveyed by DAMPs, which include exposure of calreticulin on the cell surface and the release of HMGB1 and ATP [39]. In the current study, A431 and BT-474 cells treated with NIR-PIT showed disappearance of HMGB1 from the nucleus and an increase in extracellular ATP at all concentrations. These results suggest that NIR-PIT efficiently triggered ICD. In contrast, the disappearance of HMGB1 and the increase in extracellular ATP in A431 cells were observed only at 50 µM TS-PDT, while BT-474 cells did not show any translocation/release of DAMPs under the same conditions. These results suggest that TS-PDT can trigger ICD, but its efficiency varies between cell lines, even when treated under the same conditions. Thus, the induction of DAMPs by TS-PDT was observed only at 50 µM and was less efficient than that of NIR-PIT, which relies on antibody-binding to proteins expressed on the cell membrane.

This study has several limitations. First, the effects of the light irradiation conditions on the patterns of cell death and the induction of DAMPs were not examined. NIR-PIT and PDT were performed in combination with the infusion of photoreactive agents and light irradiation; thus, irradiation conditions were also involved in the therapeutic effects. In the future, to understand the characteristics of the two treatment modalities in more detail, it will be necessary to analyze the involvement of irradiation conditions. Furthermore, cytotoxic effects and cell death pathways could be more unambiguously assessed by means of clonogenic assay and subcellular localization analysis. Second, the in vivo effects of NIR-PIT and PDT were not compared in this study. An in vivo follow-up study is important in terms of characterizing cellular affinity and cell death, which were examined in vitro in the present study. Furthermore, in vivo TS-PDT causes tumor cell death indirectly by damaging endothelial cells in the vascular system, which interrupts the nutritional supply to tumors [33]. However, it was reported that NIR-PIT may also cause vascular occlusion [40]. Thus, NIR-PIT and PDT might have direct and indirect effects; therefore, it is necessary to comprehensively compare the characteristics of these therapies using animal tumor models.

## 5. Conclusions

Here, we report a comparative study of NIR-PIT and TS-PDT in vitro (Table S1). mAb-IR700 is a molecule-selective agent, and NIR-PIT induces necrosis. In addition, NIR-PIT induces translocation/release of DAMPs, which was consistent regardless of the concentration or cell line used. However, talaporfin sodium was taken up into cells irrespective of cell type, and when TS-PDT induced concentration-dependent cell death, the patterns of cell death shifted from apoptosis to necrosis. For TS-PDT, the induction of DAMPs occurred only at the highest concentration, and the sensitivity was dependent on the cell line used. NIR-PIT, which relies on the binding of antibodies to proteins expressed on the cell membrane, was more efficient in terms of tumor visualization and induction of DAMPs than TS-PDT. To determine the usefulness of the two therapies in more detail, further in vivo follow-up studies based on our in vitro results are necessary.

## Data Availability

The data presented in this study are available on request from the corresponding author.

## Supplementary Materials

The following supporting information can be downloaded at: https://www.mdpi.com/article/10.3390/cancers15133400/s1. Figure S1. Binding/uptake specificity and cellular distribution. (A) Quantification of cellular binding/uptake of mAb-IR700 and talaporfin sodium. (B) Fluorescence images for two mAb-IR700 forms (targeting EGFR or HER2) in three cell lines. Live cells were stained for nuclei (blue) after incubation with mAb-IR700 or talaporfin sodium (magenta). Scale bar = 20 µm. Figure S2. Time course images during irradiation in BALB/3T3 cells. Bright field images of BALB/3T3 cells. 0 min: before irradiation; 4 min: immediately after irradiation; 15 min: approximately 10 min after irradiation. White arrow indicates blebbing. Scale bar = 20 µm. Figure S3. Calcein-AM/PI double-staining in A431 cells. At the highest dose of NIR-PIT or TS-PDT, calcein fluorescence decreased after 30 min, although PI fluorescence was undetected at all time points observed. The bottom panel shows images of PI-positive dead cells as a positive control of PI staining. White arrow indicates blebbing. Scale bar = 20 µm. Figure S4. The procedure of ROIs setting and the representative images with ROIs in HMGB1 translocation assessment. Nuclei ROIs were set in NucBlue images. Then, the ROIs were applied to HMGB1 images. Nuclei that were not fully in the field-of-view and nuclei in mitotic phase (white arrow) were excluded from the assessment. Figure S5. The structure of agents used in the study. (A) mAb-IR700. (B) talaporfin sodium. Table S1. Properties of NIR-PIT and TS-PDT in this study.
