# Supplementary material for: In Vitro Comparative Study of Near-Infrared Photoimmunotherapy and Photodynamic Therapy
Source: Cancers (Basel). 2023 Jun 28;15(13):3400. doi: 10.3390/cancers15133400 (PMC10340659; doi:10.3390/cancers15133400)
Supplement: Supplementary file 1 [file cancers-15-03400-s001.zip › cancers-2423805-supplementary.pdf]

Type of the Paper (Article.)

Supplementary Materials; In vitro comparative study of photoimmunotherapy and photodynamic therapy

Susumu Yamashita, Miho Kojima, Nobuhiko Onda and Makoto Shibutani

The Supporting Information includes:

Supporting Information Materials and Methods

Supporting Information Figure S1–5

Supporting Information Tables S1

Corresponding authors: Makoto Shibutani (mshibuta@cc.tuat.ac.jp)

Supporting Information Materials and Methods

HMGB1 translocation assessment

HMGB1 translocation was assessed by measuring the mean fluorescence intensity of nuclear HMGB1. The experiment was performed twice in duplicate samples. The assessment of HMGB1 immunofluorescence images was performed by Image J software. The images with at least 20 cells after excluding mitotic cells in each field-of-view were used. To set Region of interests (ROIs) of nuclei area, NucBlue staining images was used. Then, the ROIs were applied for HMGB1 immunofluorescence images in the same field-of-view as the NucBlue stained images. Statistical analysis was performed on the mean fluorescence intensity of nuclear HMGB1 from a total of five fields-of-view obtained. The representative images with ROIs are provided in Figure S4.

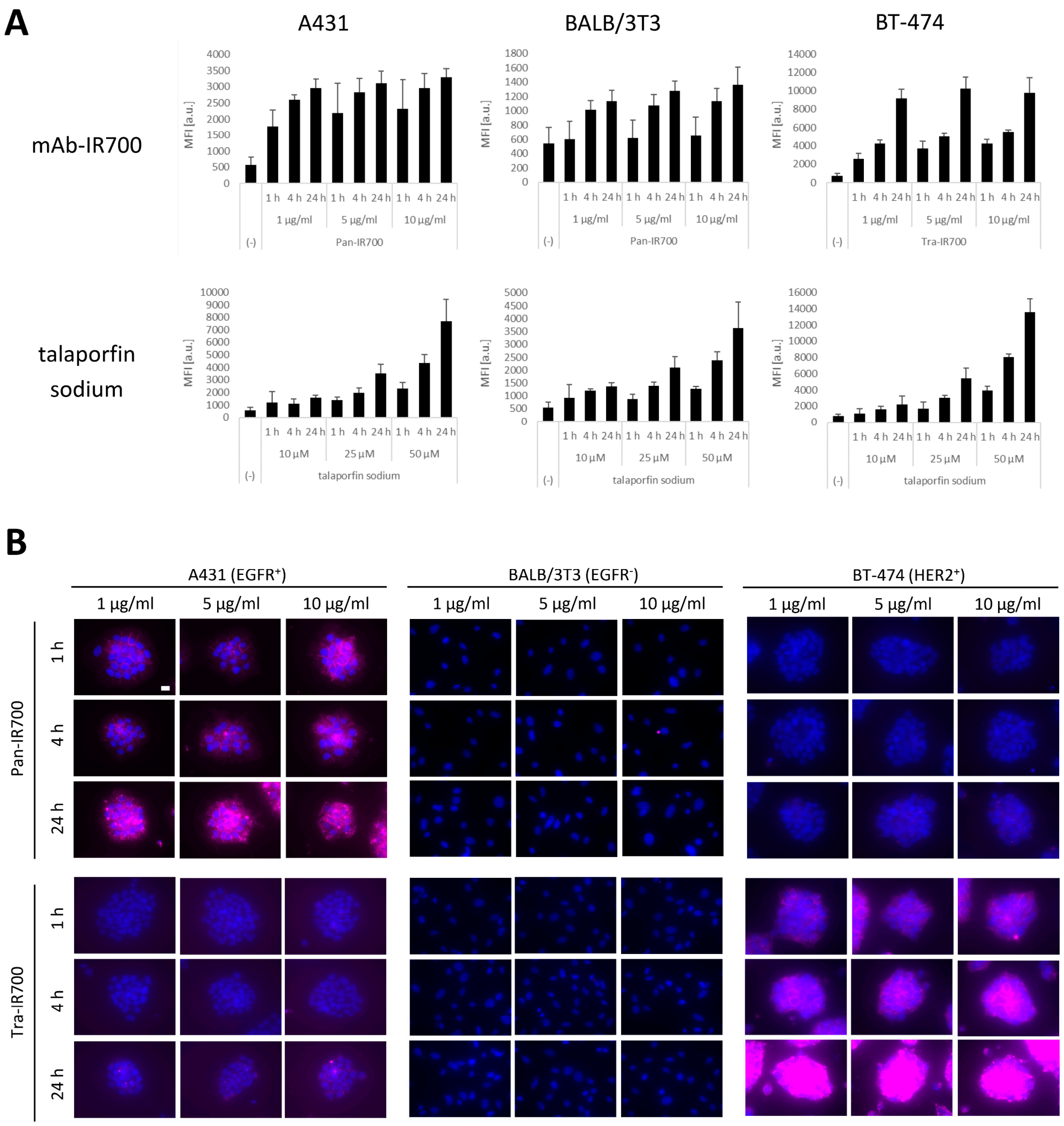

Figure S1. Binding/uptake specificity and cellular distribution. (A) Quantification of cellular binding/uptake of mAb-IR700 and talaporfin sodium. (B) Fluorescence images for two mAb-IR700 forms (targeting EGFR or HER2) in three cell lines. Live cells were stained for nuclei (blue) after incubation with mAb-IR700 or talaporfin sodium (magenta). Scale bar = 20 µm.

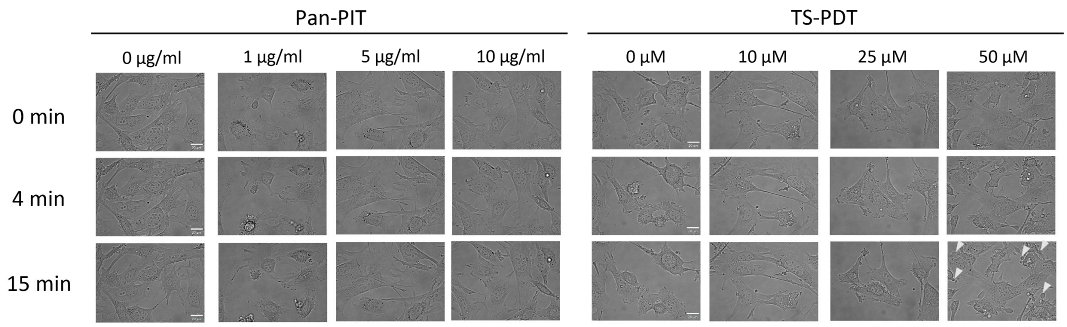

Figure S2. Time course images during irradiation in BALB/3T3 cells. Bright field images of BALB/3T3 cells. 0 min: before irradiation; 4 min: immediately after irradiation; 15 min: approximately 10 min after irradiation. White arrow indicates blebbing. Scale bar = 20 μm.

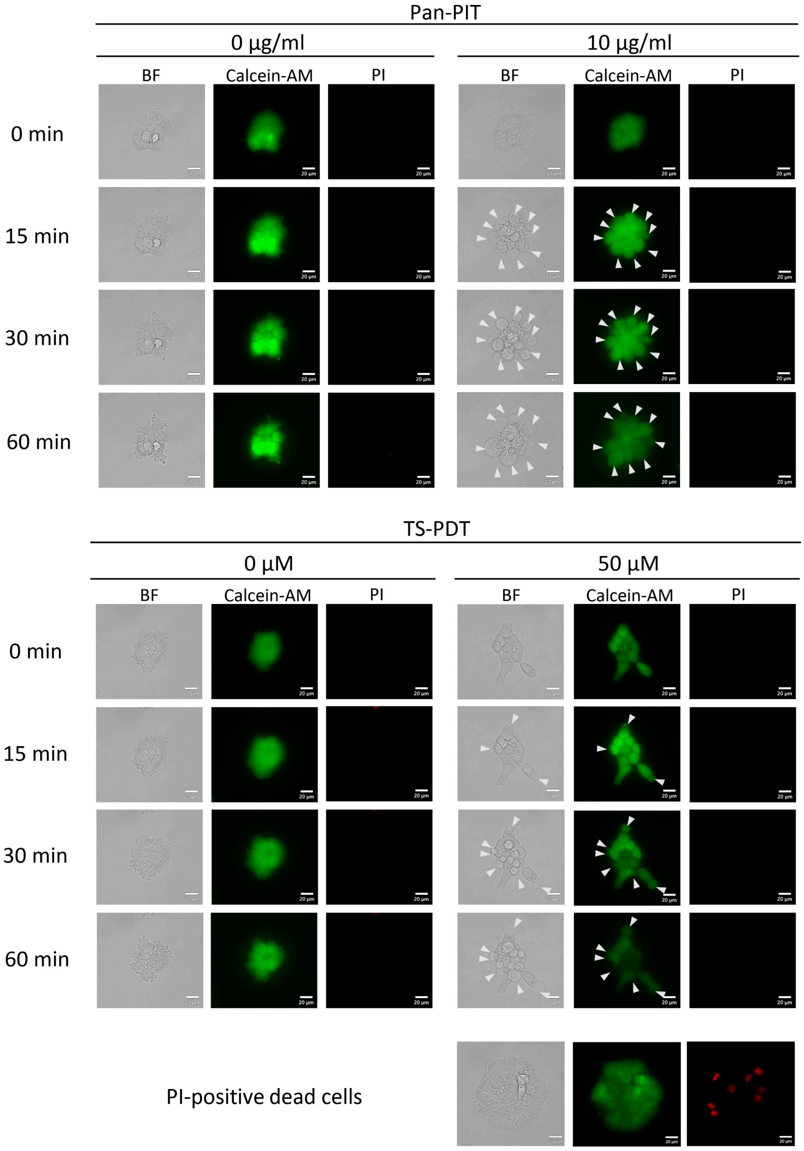

Figure S3. Calcein-AM/PI double-staining in A431 cells. At the highest dose of NIR-PIT or TS-PDT, calcein fluorescence decreased after 30 min, although PI fluorescence was undetected at all time points observed. The bottom panel shows images of PI-positive dead cells as a positive control of PI staining. White arrow indicates blebbing. Scale bar = 20 µm.

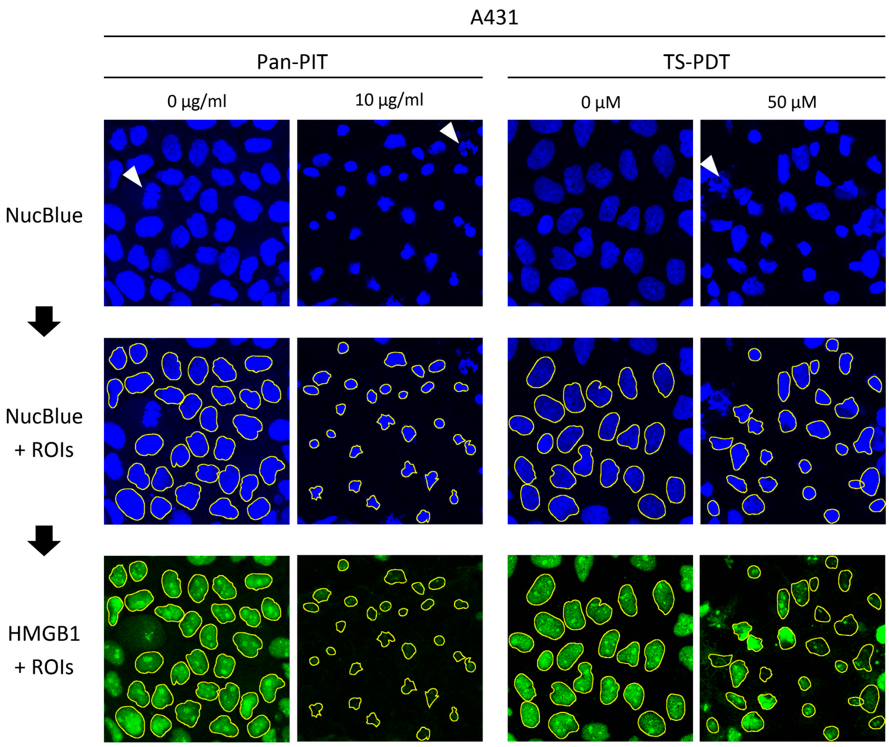

Figure S4. The procedure of ROIs setting and the representative images with ROIs in HMGB1 translocation assessment. Nuclei ROIs were set in NucBlue images. Then, the ROIs were applied to HMGB1 images. Nuclei that were not fully in the field-of-view and nuclei in mitotic phase (white arrow) were excluded from the assessment.

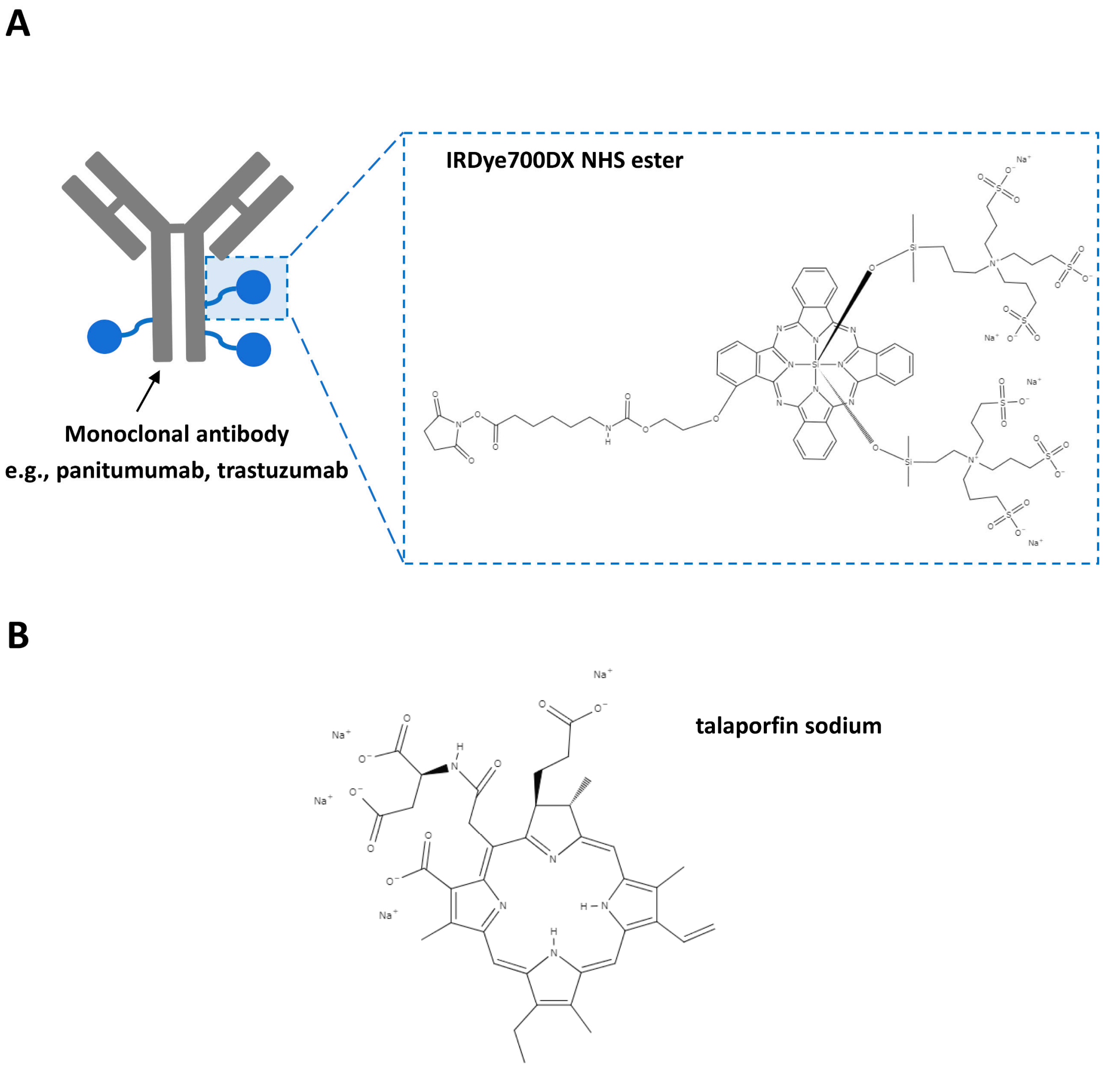

Figure S5. The structure of agents used in the study. (A) mAb-IR700. (B) talaporfin sodium.

Table S1. Properties of NIR-PIT and TS-PDT in this study.

Properties	NIR-PIT	TS-PDT
Cell specificity	Molecule-specific	Non-selective cell type
Efficacy of tumor cell visualization (Lower limit of concentration)	≥ 1 µg/ml (≈ 6.8 nM)	≥ 25 µM
Efficacy of tumor cell death (Lower limit of concentration)	≥ 6.8 nM	≥ 25 µM
Pattern of cell death	Necrosis (6.8–68 nM)	Apoptosis (25 µM), Necrosis (50 µM)
Appearance of DAMPs	≥ 6.8 nM	50 µM
